# Spontaneous cystic duct perforation mimicking intestinal obstruction in a 7-month-old infant: a case report

**DOI:** 10.1097/RC9.0000000000001066

**Published:** 2026-09-30

**Authors:** Mostafa Abrishami, Ahmad Mohammadipour, Afsaneh Hokmabadi, Marjan Joudi, Amirhossein Sarsarabi, Khashayar Atqiaee

**Affiliations:** a Department of Pediatric Surgery, Faculty of Medicine, Mashhad University of Medical Sciences, Mashhad, Iran; b Department of Anatomical Sciences and Cell Biology, Mashhad University of Medical Sciences, Mashhad, Iran

**Keywords:** biliary tract perforation, cystic duct, infant, intraoperative cholangiography, peritonitis

## Abstract

**Introduction:**

Spontaneous extrahepatic biliary perforation is rare during infancy, with isolated cystic duct perforation representing an exceedingly uncommon entity. The literature lacks standardized diagnostic pathways for atypical presentations, often delaying recognition. We report a case of a 7-month-old infant presenting with features mimicking mechanical bowel obstruction, highlighting the diagnostic gap and the need for high clinical suspicion.

**Case presentation:**

A 7-month-old boy presented with intermittent vomiting, constipation, and mild abdominal distension. Initial ultrasound revealed free peritoneal fluid and clustered ileal loops. Despite transient conservative improvement, localized right upper quadrant tenderness worsened by day 4. Repeat ultrasound demonstrated increased fluid, gallbladder distension with sludge, and a blind-ending subhepatic structure. Exploratory laparotomy identified bilious ascites and a perforation at the cystic duct–common bile duct junction containing an intraluminal calculus. Intraoperative cholangiography confirmed distal ductal patency without pancreaticobiliary maljunction. Cholecystectomy with primary ductal repair was performed. Recovery was uneventful at the 3-month follow-up.

**Conclusion:**

Cystic duct perforation can mimic intestinal obstruction in the absence of jaundice. A multifactorial etiology involving transient stone impaction, chronic inflammation, and ductal wall weakness is proposed. Intraoperative cholangiography is mandatory to guide surgical repair and exclude distal obstruction. Standardized long-term surveillance is recommended to monitor for potential ductal stricture formation.

## Introduction

Spontaneous biliary perforation (SBP) in infancy is a rare but life-threatening cause of biliary peritonitis and acute abdomen. It is defined as non-traumatic leakage of bile from the extrahepatic biliary tree into the peritoneal cavity, most frequently localized to the anterior wall of the common bile duct (CBD) or common hepatic duct near the junction with the cystic duct. Isolated cystic duct perforation represents an exceedingly rare subset^[^1–3^]^. Despite its gravity, evidence-based management protocols remain limited due to the scarcity of reported cases, particularly concerning this isolated variant. This report aims to bridge that knowledge gap by detailing a presentation that underscores the necessity for a high index of clinical suspicion when managing atypical infantile abdominal emergencies.

HIGHLIGHTS

- Isolated cystic duct perforation is exceptionally rare in infancy, accounting for less than ˂10% of spontaneous biliary perforations.
- The condition’s nonspecific presentation – mimicking common surgical emergencies such as intestinal obstruction – may lead to a delayed diagnosis.
- Clinical suspicion for biliary pathology must remain high in infants presenting with unexplained ascites, even in the absence of jaundice.
- Intraoperative cholangiography is critical for definitive anatomical delineation and surgical decision-making.
- Primary repair with cystic duct ligation is a viable, effective management strategy with favorable short-term outcomes.

Diagnosis is challenging because clinical manifestations in early infancy are typically insidious and nonspecific. Infants frequently present with progressive abdominal distension, bilious or non-bilious vomiting, constipation or obstipation, fluctuating ascites, fever, and irritability, thereby mimicking more common pediatric gastrointestinal conditions such as intussusception, incarcerated hernia, sepsis, or mechanical bowel obstruction. Although fluctuating jaundice and acholic stools are considered classic features, their absence in a notable proportion of patients frequently misleads clinicians and delays definitive intervention^[^1,3,4^]^.

The underlying pathogenesis remains incompletely understood and is likely multifactorial. Specifically, the interplay between congenital ductal fragility – such as focal wall weakness – and acquired inflammatory triggers, including biliary calculi, transient mechanical obstruction, and physiological stress, remains an area requiring further clinical investigation to effectively differentiate it from underlying syndromic associations such as biliary maljunction. Intraoperative cholangiography (IOC) remains the gold standard for defining anatomical integrity, excluding distal obstruction, and tailoring surgical repair^[^1–6^]^.

We report a rare case of isolated cystic duct perforation in a 7-month-old infant who presented with vomiting, obstipation, and abdominal distension mimicking mechanical bowel obstruction. This report provides clinical insights into diagnostic red flags for biliary etiology in non-jaundiced infants and emphasizes the imperative for standardized surgical management. This case report was prepared in accordance with the updated 2025 SCARE criteria^[^7^]^.

## Case presentation

A previously healthy 7-month-old male infant presented to the emergency department with a 2-day history of intermittent bilious vomiting, irritability, and obstipation. Perinatal history was uneventful. His surgical history was notable for an uncomplicated bilateral open inguinal herniorrhaphy performed at 4 months of age. While this procedure was routine, we postulate that the perioperative physiological stressors, including fasting, anesthesia, and transient dehydration, may have promoted biliary stasis, thereby contributing to the subsequent precipitation of biliary sludge and eventual lithiasis. Nutritional history included exclusive breastfeeding for the first 4 months, followed by the introduction of age-appropriate complementary foods.

On admission, the infant was alert and hemodynamically stable (heart rate 128 beats/min, respiratory rate 32 breaths/min, body temperature 36.8°C). Physical examination revealed mild, generalized abdominal distension without localized tenderness, guarding, or palpable masses. Initial laboratory testing demonstrated leukocytosis [white blood cell count (WBC) of 17.9 × 10^9^/L; 68% neutrophils], normal serum electrolytes, normal renal function, and normal baseline serum bilirubin and transaminases (see Table 1 for the clinical timeline). Supine abdominal radiography revealed mild gaseous distension of bowel loops with retained fecal matter but no pneumoperitoneum or classic mechanical obstruction patterns (Fig. 1). Initial abdominal ultrasonography demonstrated moderate free peritoneal fluid and clustered distal small-bowel loops with a blind-ending tubular structure, leading to a presumptive diagnosis of bowel obstruction secondary to post-surgical adhesions or an inflamed Meckel diverticulum.

**Figure 1.**
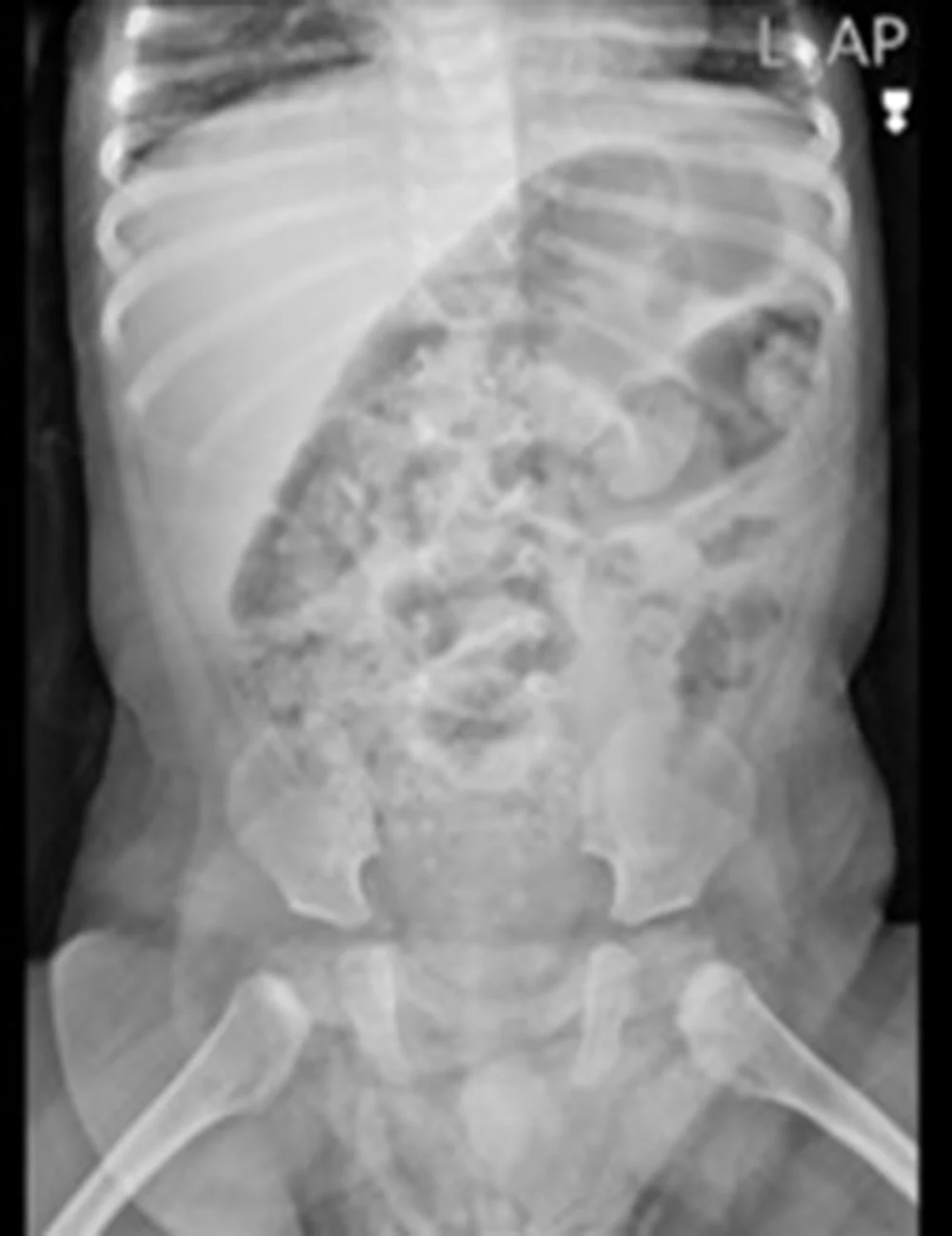
Plain supine abdominal radiograph of the 7-month-old infant, demonstrating fecal material within the colon and mild gaseous bowel distension. There is no radiographic evidence of pneumoperitoneum or definite mechanical bowel obstruction.

**Table 1:** Clinical timeline, key investigations, and interventions.

Timeline	Clinical events, investigations, and interventions
History	Bilateral inguinal hernia repair at 4 months; uncomplicated.
Day −2	Onset of intermittent bilious vomiting and obstipation.
Day 1 (admission)	Mild dehydration and abdominal distension; WBC 17.9 × 10˄^9^/L. Abdominal radiograph and ultrasonography showed fecal loading and trace free fluid. Management: NPO, IV fluids, enema, ceftriaxone, and metronidazole.
Day 2	Transient clinical improvement; WBC 10.3 × 10˄^9^/L.
Days 3–4	Clinical deterioration with right upper quadrant tenderness. CRP 3.1 mg/dL; amylase 130.7 U/L. Ultrasonography showed increasing ascites, gallbladder sludge, and a subhepatic blind-ending tubular structure.
Day 5 (surgery)	Exploratory laparotomy revealed 150 mL bilious ascites, a 2–3 mm cystic duct perforation, and a 3 mm calculus. Intraoperative cholangiography showed a patent common bile duct without maljunction. Cholecystectomy, cystic duct ligation, primary repair, and drain placement were performed.
POD 1–2	Breastfeeding resumed; drain output 25 mL bilious fluid.
POD 5–6	Drain output decreased to 3 mL serous fluid; drain removed.
Discharge	Discharged with a 7-day course of oral antibiotics.
Follow-up	1 week: weight 8.3 kg, no jaundice. 1 month: weight 8.9 kg, wound healed. 3 months: weight 9.8 kg, normal growth and stools, no complications.

The patient was initially managed conservatively with bowel rest, intravenous fluid resuscitation, broad-spectrum antibiotics, and a gentle saline enema, which yielded transient symptom relief. However, on hospital day 4, the infant developed a low-grade fever (38.2°C), lethargy, and localized right upper quadrant tenderness with guarding. Repeat abdominal ultrasonography revealed a marked increase in peritoneal fluid volume, gallbladder distension with dependent echogenic sludge, and a persistent localized fluid collection in the subhepatic space (Fig. 2).

**Figure 2.**
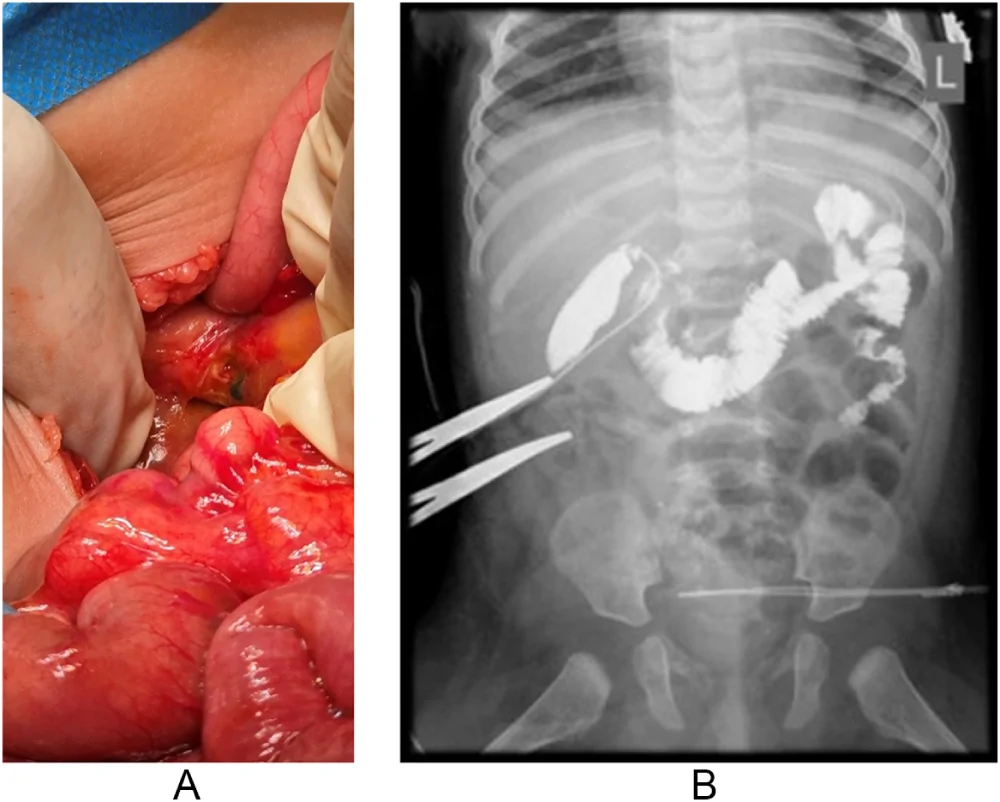
(A) Intraoperative view of a 7-month-old infant showing a 2- to 3-mm perforation of the cystic duct near its junction with the common bile duct, with an adjacent 3-mm stone and biliary ascites. (B) Intraoperative cholangiogram of a 7-month-old infant showing a patent common bile duct with free contrast passage into the duodenum and contrast extravasation at the cystic duct–common bile duct junction.

An exploratory laparotomy was performed via a right subcostal transverse incision, revealing approximately 150 mL of bilious ascites without purulent exudate. Systematic examination confirmed healthy small and large intestines, excluding bowel ischemia, perforation, or Meckel diverticulum. Inspection of the hepatobiliary triangle revealed dense inflammatory adhesions, gallbladder distension, and a 2- to 3-mm perforation at the posterior aspect of the cystic duct immediately adjacent to its confluence with the CBD. A 3-mm pigmented calculus was extracted directly from the lumen of the perforation. The focal nature of the perforation, combined with the presence of local inflammation, supports a mechanism of transient stone impaction causing localized mural necrosis, rather than a syndromic congenital ductal malformation.

IOC via direct gallbladder cannulation demonstrated contrast extravasation at the cystic duct defect, free passage of contrast into the duodenum without distal obstruction, a normal-caliber CBD, and no evidence of choledochal malformation or pancreaticobiliary maljunction. A cholecystectomy was completed, the cystic duct was divided flush with healthy tissue, and the junctional defect was closed primarily using interrupted 5–0 absorbable monofilament sutures. The peritoneal cavity was thoroughly irrigated with warm saline, and a closed-suction drain was positioned in the subhepatic space (Fig. 3).

**Figure 3.**
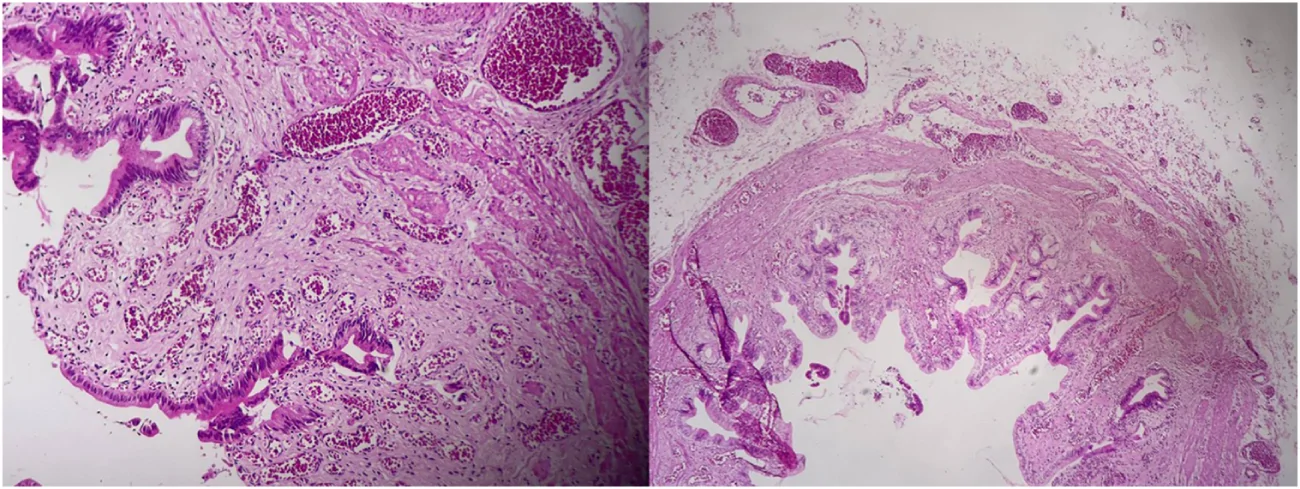
Hematoxylin and eosin–stained sections of the resected gallbladder (left, ×100; right, ×40) demonstrating mucosal folds lined by simple columnar epithelium; prominent vascular congestion with dilated capillaries and venules engorged with erythrocytes; and a mild chronic inflammatory infiltrate composed of lymphocytes and plasma cells within the muscularis, consistent with chronic cholecystitis with vascular congestion.

Histopathological examination of the gallbladder confirmed chronic cholecystitis with vascular congestion and lymphoid infiltration within the muscularis. Stone analysis confirmed a composition of calcium oxalate and bilirubin. Postoperative recovery was smooth: oral feeding resumed on postoperative day 1, drain output remained serosanguineous (<10 mL/24 h) with no biliary leakage, and the drain was safely removed on postoperative day 6. The infant was discharged on oral antibiotics. At his 3-month follow-up, he demonstrated normal developmental milestones, steady weight gain (9.8 kg), normal liver enzymes, and no recurrent gastrointestinal or biliary symptoms.

## Discussion

Spontaneous perforation of the extrahepatic biliary tree is an uncommon cause of acute abdomen in infancy, with the cystic duct being one of the least frequently reported anatomical sites. The current literature on this condition is sparse and often heterogeneous, frequently conflating cystic duct lesions with more CBD perforations. This case report specifically addresses the diagnostic challenges and anatomical nuances inherent in isolated cystic duct perforation, filling a critical gap in our understanding of how this rare entity can mimic common pediatric emergencies such as intestinal obstruction. The primary focus in evaluating this case lies in understanding the interplay among the surgical history, calculus formation, chronic cholecystitis, and potential underlying congenital or syndromic abnormalities.

### Pathophysiology specific to this case: calculi, inflammation, and herniorrhaphy

The pathogenesis of SBP in this infant appears multifactorial, representing a sequence of mechanical and inflammatory insults on a background of developmental vulnerability:

- **Calculus formation and transient obstruction**: Biliary lithiasis is uncommon in healthy infants but can develop due to bile stasis, altered bile composition, or localized infection. In this patient, stone analysis revealed calcium oxalate and pigment components. Transient impaction of this 3-mm calculus at the narrow cystic duct–CBD junction likely caused an acute rise in intrabiliary luminal pressure, local mucosal ischemia, and eventual transmural necrosis, leading to rupture^[^8–10^]^.
- **Role of chronic cholecystitis**: Histopathology confirmed established chronic cholecystitis with vascular congestion and mural infiltration. This indicates that subclinical, low-grade inflammatory episodes predated the acute perforation. Chronic inflammatory mural weakening diminishes the tensile strength of the ductal epithelium, rendering it susceptible to perforation even under modest increases in intrabiliary pressure^[^9–11^]^.
- **Relevance of prior herniorrhaphy**: The infant’s bilateral inguinal herniorrhaphy at 4 months warrants careful evaluation. While direct iatrogenic trauma to the biliary tree during inguinal surgery is anatomically impossible, the physiological stress, perioperative fasting, transient dehydration, and anesthetic agents during that previous episode may have favored biliary stasis and sludge precipitation. Furthermore, intermittent peritoneal irritation or micro-adhesions could have contributed to altered gastrointestinal motility, compounding the diagnostic dilemma when intestinal symptoms re-emerged^[^5,9,12^]^.

### Differential pathophysiology and syndromic associations

When encountering pediatric biliary perforation, several underlying congenital malformations and syndromic entities must be considered and differentiated:

- **Pancreaticobiliary maljunction (PBDJ**): An anomalous, long common channel permits continuous bidirectional reflux of pancreatic juice into the biliary tract, causing enzyme activation, chemical cholangitis, wall degradation, and spontaneous blowout^[^1,4,13^]^. In our patient, IOC definitively excluded PBDJ and a choledochal cyst.
- **Syndromic biliary anomalies**: Extrahepatic biliary fragility or ductal ectasia has been described in association with conditions such as Alagille syndrome, cystic fibrosis, and congenital hepatic fibrosis. Review of the patient’s physical features, neonatal history, cardiac evaluation, and rapid recovery ruled out these syndromic conditions^[^1,3,14^]^.
- **Developmental ductal wall weakness**: A localized developmental defect in the muscularis of the bile duct wall at the embryological junction of the cystic duct and hepatic ducts represents an area of congenital locus minoris resistentiae, which predisposes to rupture upon sudden intraductal hypertension^[^1–4^]^.

### Key diagnostic nuances and mimicry of bowel obstruction

A pivotal clinical takeaway is that SBP frequently presents without jaundice and closely mimics mechanical intestinal obstruction or peritonitis of bowel origin^[^2,4,5^]^. Bile causes chemical peritonitis that induces paralytic ileus, vomiting, abdominal distension, and obstipation. Ultrasonography can be deceptive; ascites with matted, sluggish bowel loops and peri-duodenal fluid can easily be misinterpreted as internal herniation, adhesive obstruction, or Meckel’s diverticulitis^[^15,16^]^. Clinicians must maintain a high index of suspicion: when an infant presents with evolving unexplained ascites and localized right-sided peritonitis, a hepatobiliary etiology should remain at the top of the differential^[^4,15^]^.

### Role of intraoperative cholangiography and surgical strategy

IOC remains indispensable in managing biliary perforation^[^4,10,17^]^. It serves three critical functions: (1) accurately mapping the exact site of extravasation, (2) confirming distal ductal patency into the duodenum, and (3) ruling out associated structural malformations (e.g., PBDJ, choledochal cyst, distal web/atresia). Simple drainage alone is insufficient if there is an obstructing calculus or severe local cholecystitis. Definitive cholecystectomy, extraction of the stone, primary ductal repair over healthy tissue, and subhepatic drainage provided curative treatment in this case^[^12,17–19^]^.

## Strengths and limitations

This study possesses notable clinical strengths, including detailed documentation of an exceptionally rare anatomical entity (isolated cystic duct perforation with cholelithiasis), intraoperative cholangiographic confirmation, comprehensive histopathological evaluation, and complete short-term surgical follow-up. Furthermore, the report adheres strictly to the SCARE 2025 guidelines.

However, several limitations exist. As a single case report, generalizability is limited. The exact etiology of calcium oxalate calculus formation in this previously healthy infant could not be completely elucidated due to the absence of specialized metabolic and bile composition profiling. Furthermore, long-term hepatobiliary imaging was not performed at the 3-month mark. Consequently, we suggest that future prospective multicenter studies focus on standardizing long-term surveillance protocols in pediatric biliary perforation cases to monitor for delayed ductal strictures and to better understand potential metabolic predispositions^[^5,12,14^]^.

## Clinical application and practical recommendations

The findings of this case provide several direct applications for pediatric clinical practice:

1. **Consider biliary etiology in atypical obstruction**: In infants presenting with non-specific intestinal obstruction, persistent leukocytosis, and progressive ascites – even in the complete absence of clinical jaundice – biliary tract perforation must be actively excluded.
2. **Serial ultrasound re-evaluation**: When initial imaging is equivocal, serial ultrasonography focusing on the gallbladder wall, sludge accumulation, and subhepatic fluid pooling is critical to detect evolving biliary pathology.
3. **Mandatory cholangiography**: Whenever biliary peritonitis is identified during exploratory laparotomy or laparoscopy, IOC is essential prior to definitive repair to ensure distal ductal clearance and to exclude anatomical anomalies.

## Conclusion

Isolated cystic duct perforation represents an exceptionally rare pediatric emergency that frequently mimics mechanical bowel obstruction and can present in the complete absence of jaundice. This report highlights that the underlying pathophysiology is typically multifactorial, involving an interplay between transient calculus impaction, localized chronic inflammation, and potential developmental ductal wall susceptibility. Prompt surgical exploration with routine IOC remains essential to definitively delineate the anatomy and guide the surgical strategy. Moving forward, the establishment of standardized long-term surveillance protocols is warranted to better monitor for potential delayed ductal strictures and to clarify the natural history of this rare condition. Ultimately, definitive surgical management – comprising cholecystectomy, primary ductal repair, and closed-suction drainage – achieves excellent clinical outcomes in the pediatric population.

## Data Availability

The clinical and laboratory data supporting the findings of this case report are contained within the article. Additional patient-level details are not publicly available due to privacy and confidentiality considerations but may be obtained from the corresponding author on reasonable request, subject to appropriate institutional and consent restrictions.
